# Antimicrobial Susceptibility Pattern and Serotype Distribution of Streptococcus pneumoniae Isolates From a Hospital-Based Study in Chandigarh, North India

**DOI:** 10.7759/cureus.21437

**Published:** 2022-01-19

**Authors:** Swati Sharma, Monica Sharma, Pallab Ray, Anuradha Chakraborti

**Affiliations:** 1 Experimental Medicine and Biotechnology, Postgraduate Institute of Medical Education and Research, Chandigarh, IND; 2 Microbiology, Postgraduate Institute of Medical Education and Research, Chandigarh, IND

**Keywords:** hospital-based research, vaccine, serotyping, antibiotic susceptibility, streptococcus pneumoniae

## Abstract

*Streptococcus pneumoniae *(pneumococcus) causes significant infection-related morbidity and mortality worldwide. The genome plasticity of pneumococcus is an essential factor in antibiotic resistance, serotype switching, and the emergence of nonvaccine serotypes. Information regarding the serotype distribution as well as antimicrobial susceptibility in pneumococcus clinical isolates responsible for various infections in Northern India is limited. Here, we have explored the antibiotic resistance and serotype pattern associated with *S. pneumoniae* infections from both invasive and noninvasive sites of patients of all ages, visiting out-patient department of a tertiary care hospital (PGIMER, Chandigarh, India). This study was carried out on 68 *S. pneumoniae* isolates and the isolates exhibited the highest resistance (76.5%) to cotrimaxozole followed by resistance toward tetracycline (36.8%) and erythromycin (23.5%). All isolates showed vancomycin susceptibility and 86.8% of isolates showed sensitivity to chloramphenicol. Multidrug resistance was found in 32% (n=22) of the *S. pneumoniae* isolates showing resistance toward three different antibiotics. Serotype 19F was found to be the most prevalent serotype (39%) followed by serotypes 6A/B/C (19%) and 1 (12%). These data shed light on the latest trends in antibiotic susceptibility and prevalent serotype patterns of hospital-based *S. pneumoniae* isolates. This information can be helpful in designing future disease-preventive strategies.

## Introduction

*Streptococcus pneumoniae* is responsible for causing various invasive as well as noninvasive diseases like pneumonia, bacteremia, otitis media, sepsis, and meningitis [1]. Infections caused by pneumococcus are responsible for significant morbidity and mortality worldwide. According to an SRMGBD (2016) study by Troeger C (2018) [2], *Streptococcus pneumoniae* was responsible for 1,189,937 deaths with 95% uncertainty index (690 ,445-1,770, 660). Most of the pneumococcal-related cases are reported from Asia and Africa, in which India accounts for 27% of the cases [3]. In India, the number of deaths associated with pneumococcal infections is estimated to be around 68,700 (an uncertainty range of 44,600-86,000) [4]. Emerging drug resistance in the case of pneumococcus is a serious problem that is responsible for the increased incidence of the novel, nonvaccine serotype, and multidrug-resistant strains.

Globally, a relatively high prevalence of strains resistant to β -lactams (penicillin) and macrolides has been observed [5]. More than a hundred different serotypes of pneumococcus have been identified. The pneumococcal serotype distribution has shown geographical variation globally, with lower-to-middle income countries (LMIC) and densely populated countries like India bearing the significant burden of infections and serotype variation [6,7]. The currently available vaccines exhibit limited coverage of serotypes and fail to account for the phenomenon of capsular replacement. These vaccines have also been shown to confer poor mucosal immunity and are not easily accessible to the economically poorer countries due to their high costs [8-10]. This increase in the number of novel, antibiotic-resistant strains, and the overall increase in the number of serotypes have necessitated the need for continued sentinel surveillance. Also, data on the serotypes circulating in the community have been reported but information on serotypes causing serious infections that lead to hospital visits and subsequent hospital admissions is scarce [11]. In our hospital-based study, the distribution of serotypes and the antibiotic resistance patterns of *S. pneumoniae *isolates collected from invasive and noninvasive sites is reported. This study profiled the sample-wise serotype prevalence and susceptibility trends and analyzed the vaccine coverage of the detected serotype, which is crucial to define the prevention and treatment strategies for *S. pneumoniae* infections.

## Materials and methods

Sample collection

This study was approved by the Institute’s Ethics Committee (IEC Memo no. 87770-PG11-ITRG/11384 & INT/IEC/2018/1081).* S. pneumoniae* standard/reference strains MTCC 655 (NCTC 7465), as well as D39 (NCTC 7466), were obtained from Microbial Type Culture Collection (MTCC), IMTECH, Chandigarh, India. Clinical strains from blood, cerebrospinal fluid (CSF), sputum, and nasopharynx were isolated from lower respiratory tract infection patients. The presence of *S. pneumoniae* was confirmed into all the samples via the Bacteriology unit of Department of Medical Microbiology, PGIMER Hospital, Chandigarh (Union Territory), a tertiary care center catering to the population of union territory and nearby states, and after that, those samples were collected from the Bacteriology unit of Department of Medical Microbiology. These samples were received from the out-patient department of PGIMER Hospital, Chandigarh. Isolates from the patients diagnosed with any other bacterial infection or illness or having any genetically inherited diseases were excluded.

Antibiotic susceptibility testing

All *S. pneumoniae* isolates were cultured and maintained for further susceptibility testing and evaluation of serotypes. Kirby Bauer disc diffusion technique was used for screening of all *S. pneumoniae* isolates as per Clinical and Laboratory Standard Institute (CLSI) guidelines [12,13]. The antibiotics used for screening included oxacillin (OXA; 1 µg), erythromycin (ERY; 15 µg), co-trimoxazole (SXT; 25 µg), tetracycline (TET; 30 µg), vancomycin (VAN; 30 µg), ciprofloxacin (CIP; 5 µg), cefotaxime (CTX; 30 µg), amoxicillin (AMX; 10 µg), levofloxacin (LVX; 5 µg), and chloramphenicol (CHL; 30 µg). The overnight grown cultures were spread on blood agar plates and antibiotic discs were placed at appropriate distances on the plates. The plates were incubated at 37ºC for 18 h and a zone size of each antibiotic was studied. Results were interpreted as resistant, susceptible, or intermediate according to the CLSI standards of interpretative criteria. The isolates showing a zone ≤19 mm around the OXA disk were interpreted for minimum inhibitory concentration (MIC) of penicillin by E-test (AB Biodisk). The categorical interpretations of MIC (µg/mL) were done using CLSI susceptibility breakpoints and isolates were labeled as S, I, or R to penicillin if MIC was <2.0 µg/mL, 4 µg/mL, or ≥8.0 µg/mL, respectively (“CLSI,” 2012). The isolates resistant to three or more classes of antibiotics were labeled as multi-drug resistant (MDR).

Molecular/polymerase chain reaction typing of the isolates

*S. pneumoniae* isolates were grown in 5 mL THY (OD600 nm - 0.4-0.5) and genomic DNA was extracted from the bacterial cell pellet using the cetyl-trimethylammonium bromide (CTAB) method [14]. *S. pneumoniae* isolates were typed based on the cps gene cluster using multiplex-polymerase chain reaction (PCR). All the available serotype-specific primer sequences were obtained from Centers for Disease Control and Prevention (CDC) webpage (http://www.cdc.gov/streplab/pcr.html, http://www.cdc.gov/streplab/downloads/pcr-oligonucleotide-primers.pdf) to target serotypes 1, 2, 3, 4, 5, 6A/B/C, 6C,7C/B, 7F/A, 8F, 9V/A, 9N/L, 10A, 10F/C, 11A/D, 12F, 13, 14, 15A/F, 15B/C, 16F, 17F, 18A/B/C, 19A, 19F, 22F/A, 23A, 23B, 23F, 33F/A/37, 34, 35B, 35A/C/42, 35F/47F, 38/25F, and 39. A primer pair for cpsA was included as an internal control due to its highly conserved nature in all of the characterized capsular loci [15]. The primers were grouped into six-multiplex group reactions. The reactions were performed in 25 µL reaction volumes, which contained 1× PCR buffer (20 mM Tris-HCl, pH 8.0), 200 µM dNTPs, 50 mM MgCl_2_, 2.5 U Taq DNA polymerase (Bioron-Germany), 50 picomole primers cocktail, and ~100 ng DNA. PCR was performed in GeneAmp PCR system 9700 from Applied Biosystems, USA. The PCR conditions were as follows: initial denaturation - 94°C for 5 min. This was followed by 40 cycles of denaturation - 94°C for 1 min; annealing - 50°C for 90 s; extension - 65°C for 2 min 30 s. Finally, final extension was carried out at 65°C for 7 min. The amplicons were analyzed on 2% agarose gels and compared with molecular size standards (GeneRuler-Thermo). The serotypes obtained from multiplex PCR were further confirmed by DNA sequencing.

## Results

To check the antimicrobial susceptibility and serotype distribution, samples like blood (48), CSF (2), sputum (6), and nasopharynx (12) of patients suffering from lower respiratory tract infection were included in this study.

Antibiotic susceptibility

**Figure 1:**
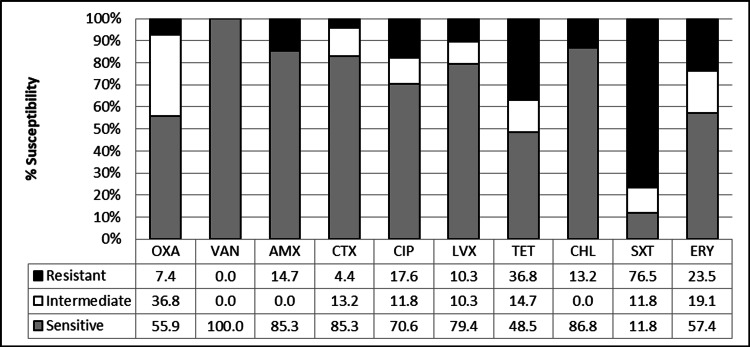
Antibiotic susceptibility pattern of pneumococcal isolates isolated from clinical samples. OXA, oxacillin; ERY, erythromycin; SXT, co-trimoxazole; TET, tetracycline; AMX, amoxicillin; VAN, vancomycin; CHL, chloramphenicol; CIP, ciprofloxacin; CTX, cefotaxime; LVX, levofloxacin.

The in vitro susceptibility of pneumococcal isolates was checked against 10 antimicrobial agents to assess their susceptibility patterns (Figure 1). Fifty-nine isolates showed antibiotic resistance to at least one of the tested antimicrobials while three were completely susceptible to tested antimicrobials including one standard strain. MIC of these isolates against benzylpenicillin E-strips ranged from 0.125 to 12 µg/mL. Penicillin resistance was detected in only five pneumococcal isolates, while the rest of the isolates showed penicillin susceptibility with MIC ≤2.0 µg/mL. Susceptibility profiling of isolates showed maximum resistance to SXT (76.5%) and complete sensitivity to VAN (100%). Besides this, many of the isolates also showed resistance to TET (36.8%) followed by ERY (23.5%) and CIP (17.6%).

The antibiotic susceptibility patterns were further analyzed on the basis of invasive and noninvasive sources of* S. pneumoniae* isolates (Table 1). All nasopharyngeal (NP) isolates were resistant to SXT, whereas only 68.8% (n=50) of the blood isolates were resistant to it. The resistance to ERY was highest (50%; n=6) in NP isolates. Sputum isolates showed maximum resistance to SXT (83%; n=5), CIP, and TET (66.6%; n=4). Ten isolates (nine blood and one NP) showed resistance for AMX and three isolates from blood were found to be resistant to CTX. The resistance to LVX was observed in three sputum as well as three blood isolates and a single isolate from NP. Likewise, CHL resistance was also detected in only seven blood and two sputum isolates. Interestingly, isolates from CSF consistently exhibited susceptibility for AMX, CTX, CHL, and LVX.

**Table 1:** Antibiotic resistance (R) and sensitive (S) pattern of pneumococcal isolates isolated from clinical samples. Blood (n=48); CSF (n=2); NP (n=12); sputum (n=6). CSF, cerebrospinal fluid; NP, nasopharyngeal.

Antibiotic names		Invasive (blood and CSF)				Noninvasive (NP and sputum)			
		Blood		CSF		NP		Sputum	
		R	S	R	S	R	S	R	S
1	Oxacillin	3	28	0	2	2	4	0	4
2	Vancomycin	0	48	0	2	0	12	0	6
3	Amoxicillin	9	38	0	2	1	11	0	6
4	Cefotaxime	3	36	0	2	0	12	0	6
5	Ciprofloxacin	7	35	0	2	1	9	4	2
6	Levofloxacin	3	38	0	2	1	11	3	3
7	Tetracycline	17	28	1	1	4	4	4	0
8	Chloramphenicol	7	41	0	2	0	12	2	4
9	Co-trimaxozole	33	8	2	0	12	0	4	0
10	Erythromycin	10	28	0	2	6	6	0	3

Occurrence of intermediate phenotype among the isolates signifies an opportunity to develop complete resistance against antibiotics, and in our study isolates, we observed a variable frequency of intermediate resistance. The isolates showed the highest intermediate resistance for oxacilin (36.8 %; n=25) while 14.7% of the isolates showed Intermediate resistance against TET (n=10), with the highest occurrence seen among NP isolates (n=4). Intermediate resistance toward CIP was observed in 16.7% NP isolates. Further, intermediate resistance toward SXT (11.8%; n=8) and ERY (19.1%; n=13) was observed among strains isolated from different sources. Blood isolates did not show any intermediates for AMX and CHL. NP and sputum isolates did not show any intermediate resistance to VAN, AMX, CTX, and LVX. One CSF isolate exhibited intermediate resistance toward TET. However, intermediate resistance to AMX and CHL was not seen in any of the isolates. When studying the susceptibility of the isolates as per the different classes of antibiotics, the highest resistance was observed against sulfonamide (76.5%) and the least resistance was observed against β-lactams (6.6%). Almost all isolates showed sensitivity toward CHL (86.8%), followed by β-lactams (80.88%), quinolones (75%), macrolides (57.4%), and TET (48.5%). The highest ratio of intermediates was observed in macrolides and TET. No intermediate resistance was observed in CHL. Further, isolates showing resistance to three or more classes of antibiotics were labeled as MDR. Multidrug resistance was observed in 32% (n=22) isolates, 96% of these MDR isolates showed resistance to SXT. The highest susceptibility was showed by β-lactams class of antibiotic VAN (100%).

Serotypes of *S. pneumoniae* isolates

**Figure 2:**
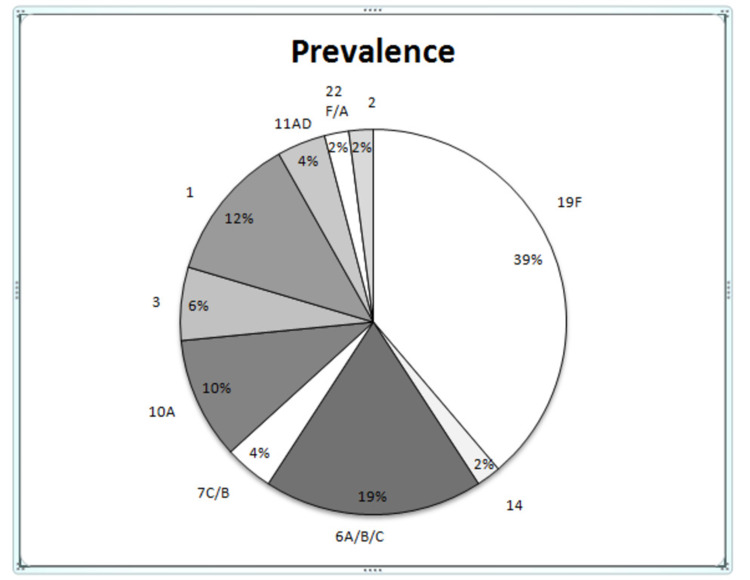
Profiling of serotypes trends in Streptococcus pneumoniae clinical isolates.

A total of six different serotypes/groups were found to be distributed among pneumococcal isolates. Serotyping was done for 68 isolates (Figure 2) in which 49 isolates were typed against 36 available primers of known serotypes and 19 isolates remain untyped due to the non-availability of primers of the remaining serotypes.

**Figure 3:**
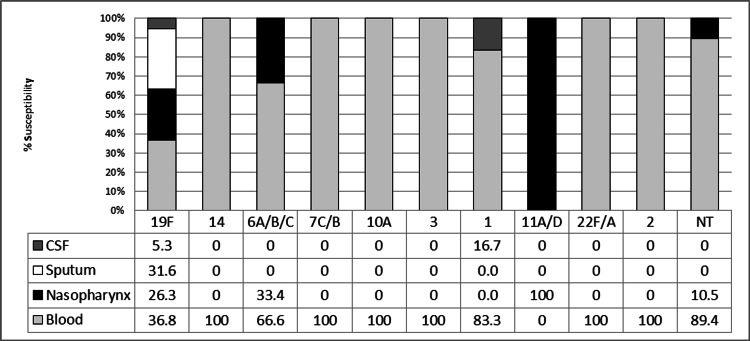
Serotype distribution of Streptococcus pneumoniae isolates from different sources. CSF, cerebrospinal fluid.

Serotype 19F was predominant in 39% (n=19) isolates followed by serotypes 6A/B/C (19%; n=9), 1 (12%; n=6), and 10A (10%; n=5) among the typed isolates. Other serogroups that were identified included 3, 7C/B, 11A/D, 14, 2, and 22F/A (in descending order of prevalence). Serogroup 11A/D was not found among blood isolates. The sequence typing conformed to the PCR serotypes and further resolved serogroup 6A/B/C and 7C/B into serotypes 6A and 7C, respectively. Blood isolates showed maximum variability in serotypes representing nine different serotypes/serogroup 19F, 14, 6A/B/C, 7C/B, 10A, 3, 1, 22F/A, and 2. All the sputum isolates showed 19F serotype, whereas CSF isolates belonged to serotype 19F and 1. Further, NP isolates were grouped under 19F, 6A/B/C, and 11A/D serotypes (Figure 3).

Sequencing at cps loci in 10 randomly chosen isolates covering seven different serotypes further confirmed the serotypes detected in the multiplex PCR method. The obtained sequence-typing nucleotide data were analyzed for homology using BLAST and hits with the highest bit score were compared with PCR-based serotypes. All sequence-derived serotypes matched exactly with PCR serotypes as per criteria mentioned elsewhere [16]. One isolate of each serogroup 6A/B/C and 7C/B was further resolved into serotypes 6A and 7C, respectively. The rest of the isolates bearing 11A/D, 22F/A, and 7C/B serogroups showed consistent outcomes upon sequence typing.

Assessment of vaccine coverage of serotypes

Analysis of the detected serotypes among clinical strains of pneumococcus for the percentage serotype coverage of various commercially vaccines (Table 2) showed that the PPSV 23 vaccine offered maximum coverage (96%) followed by PCV11 and PCV 13 (77.5%) and PCV 7, PCV 9, and PCV 10 (59.1%) [17,18].

**Table 2:** Percentage coverage of study serotypes in various pneumococcal vaccines.

Serotype of *Streptococcus pneumoniae*	Frequency in studied clinical isolates	Pneumoccocal vaccine					
		PCV 7	PCV 9	PCV 10	PCV 11	PCV 13	PPSV 23
1	6	X	✓	✓	✓	✓	✓
2	1	X	X	X	X	X	✓
3	3	X	X	X	✓	✓	✓
6A/B/C	9	✓	✓	✓	✓	✓	✓
7C/B	2	X	X	X	X	X	X
10A	5	X	X	X	X	X	✓
11A/D	2	X	X	X	X	X	✓
14	1	✓	✓	✓	✓	✓	✓
19F	19	✓	✓	✓	✓	✓	✓
22F/A	1	X	X	X	X	X	✓
Total	49	29	29	29	38	38	47
Percentage covered by vaccine		59.1	59.1	59.1	77.5	77.5	96

## Discussion

*S. pneumoniae* frequently colonizes the upper respiratory tract in humans and is responsible for causing diseases that can be minor, to life-threatening diseases like pneumonia, sepsis, and meningitis under appropriate conditions [19]. Despite the accessibility of antibiotics and the development of many vaccines, the burden of pneumococcal infections in many LMIC including India still remains high and no concrete preventive strategies have been adopted so far [7]. The genome plasticity of *S. pneumoniae* and the uneven global prevalence of serotypes limit the success of vaccines [20] and have also resulted in the development of high resistance to commonly used antibiotics. Further, immunological and economical reasons restrict the effective management of pneumococcal infections. Therefore, it is imperative to study the locally prevalent serotypes and susceptibility trends in clinical isolates that are driving pneumococcal infections. *S. pneumoniae* isolate used in this study broadly represents the noninvasive (NP and sputum) and invasive (blood and CSF) pneumococcal infections. The data obtained in this hospital-based study demonstrate maximum resistance to SXT and TET. This is supported by earlier reports from India, which also stated high resistance to these antibiotics in invasive and carrier strains [21-23]. Importantly, the resistance to these antibiotics has been found to be higher in Indian communities in comparison to other global populations [24]. Besides, resistance to antibiotics like ERY is also concerning as it is broadly used for the treatment of acute respiratory infections. We have observed its higher resistance rate in colonizer NP strains. This was also correlated in various other studies carried out in India [23,24]. In relation to the different antibiotic classes, maximum resistance was observed in sulfonamide (76.5%) followed by TET and macrolides. These results are uniform with respect to other similar studies [17,18,23]. In context, in this study, no variation in the antibiotic susceptibility trends was exhibited by the clinical isolates when compared to the previously reported results from different parts of India. The prevalence of *S. pneumoniae *serotypes affects the choice of preventive measures utilized, which makes serotyping an absolute necessity. Hence, we used a multiplex-PCR typing approach for the serotyping of isolates and its accuracy was ensured by sequence typing. We found the predominance of serotype 19F among the isolates, which was followed by serotypes 6A/B/C, 1, 10A, 3, 7C/B, 11A/D, 14, 2, and 22F/A. Prevalence of 19F was also noted in a recent study carried out in Asian countries [5]. Various studies have also reported serotype 6 as most predominant in different hospital as well as day-care setups [21,22]. Earlier, invasive bacterial infection surveillance also implicated 1 and 19 as the most invasive disease-causing serotypes [25]. In Asia-pacific region, serotypes 19F along with 23F, 19A, 14, and 6B have been shown as the most prevalent serotypes in clinical pneumococcal isolates by ANSORP, and serotype 19F and 14 were depicted as invasive pneumococcal isolates [26]. PCV10 (1,4, 5, 6B, 7F, 9V, 14, 19F, 18C, and 23F) and PCV13 (1, 3,4, 5, 6A, 6B, 7F, 9V, 14, 19A, 19F, 18C, and 23F ) are the two most widely used vaccines as recommended by WHO. Correlating the obtained serotypes against the currently used vaccines for serotypes coverage, it was found that PPSV23 (1, 2, 3, 4, 5, 6B, 7F, 9V, 10A, 11A, 12F, 14, 15B, 17F, 19A, 19F, 18C 20, 22F, and 23F) [16,18] covered 96% of the prevalent serotype followed by PCV13 (77.5%). This vaccine has been shown to provide coverage against serotypes like 19F, 14, and 23F, which are becoming increasingly resistant to antibiotics [18]. These findings can be correlated to our study as serotypes like 19F, 6B, and 3 were also found among our isolates. Further, the high mortality rates have been associated with infection of *S. pneumoniae *of serotypes 3, 6B, and 19F [27,28]. Our study has few limitations in terms of the number of isolates profiled. The number of strains included in this study is directly impacted by the inclusion and exclusion criteria. As per our exclusion criteria, we excluded patients diagnosed with any other bacterial infection or illness or a genetically inherited disease. A proportion (19%) of serotypes in this study were labeled as untyped as the current PCR-based method limits the range of serotypes (38 serogroups) as that can be detected. The use of conventional serotyping methods such as Quellung serotyping remains expensive [29]. Untyped isolates, if resolved, may help us detect more variation in the distribution of serotypes. Finally, we can say that this study contributes to our understanding of the recent antibiotic susceptibility and serotype patterns in a North Indian Union Territory. Further, this information can potentially help us in the development of better therapeutic and management strategies against pneumococcal infections.

## Conclusions

This study highlighted the antibiotic susceptibility and serotype distribution of *S. pneumoniae* isolates from PGIMER, Chandigarh, India. We observed maximum resistance in sulfonamide class of antibiotic cotrimaxazole (76.5%). Substantial resistance toward TET and macrolide antibiotics was also observed. On the other hand, maximum sensitivity was observed in beta-lactam class of antibiotics like VAN (100%) followed by CHL (86.8%). Our serotype data show 19F as the most prevalent serotype followed by 6A/B/C and 1. These are predominantly found in invasive pneumococcal diseases. Thus, this study sheds light on the present North Indian scenario regarding antimicrobial susceptibility and serotype distribution of *S. pneumoniae. *Continuous surveillance of these factors will be useful in formulating future therapeutic and preventive strategies against *S. pneumoniae.*
